# Factors Mediating Environmental Biofilm Formation by *Legionella pneumophila*

**DOI:** 10.3389/fcimb.2018.00038

**Published:** 2018-02-27

**Authors:** Arwa Abu Khweek, Amal O. Amer

**Affiliations:** ^1^Department of Biology and Biochemistry, Birzeit University, West Bank, Palestine; ^2^Department of Microbial Infection and Immunity, Center for Microbial Interface Biology, College of Medicine, Ohio State University, Columbus, OH, United States

**Keywords:** *Legionella pneumophila*, biofilm, Legionellosis, protozoa, planktonic

## Abstract

*Legionella pneumophila* (*L. pneumophila*) is an opportunistic waterborne pathogen and the causative agent for Legionnaires' disease, which is transmitted to humans via inhalation of contaminated water droplets. The bacterium is able to colonize a variety of man-made water systems such as cooling towers, spas, and dental lines and is widely distributed in multiple niches, including several species of protozoa In addition to survival in planktonic phase, *L. pneumophila* is able to survive and persist within multi-species biofilms that cover surfaces within water systems. Biofilm formation by *L. pneumophila* is advantageous for the pathogen as it leads to persistence, spread, resistance to treatments and an increase in virulence of this bacterium. Furthermore, Legionellosis outbreaks have been associated with the presence of *L. pneumophila* in biofilms, even after the extensive chemical and physical treatments. In the microbial consortium-containing *L. pneumophila* among other organisms, several factors either positively or negatively regulate the presence and persistence of *L. pneumophila* in this bacterial community. Biofilm-forming *L. pneumophila* is of a major importance to public health and have impact on the medical and industrial sectors. Indeed, prevention and removal protocols of *L. pneumophila* as well as diagnosis and hospitalization of patients infected with this bacteria cost governments billions of dollars. Therefore, understanding the biological and environmental factors that contribute to persistence and physiological adaptation in biofilms can be detrimental to eradicate and prevent the transmission of *L. pneumophila*. In this review, we focus on various factors that contribute to persistence of *L. pneumophila* within the biofilm consortium, the advantages that the bacteria gain from surviving in biofilms, genes and gene regulation during biofilm formation and finally challenges related to biofilm resistance to biocides and *anti-Legionella* treatments.

## Introduction

*Legionella pneumophila*, the causative agent of Legionellosis, was recognized as being pathogenic to humans for the first time after an outbreak of acute pneumonia at a convention of the American Legion in Philadelphia, USA in July 1976 (Fraser et al., 1977). *Legionella* can cause two clinical syndromes in humans, Legionnaires' disease (LD), a severe form of pneumonia, and Pontiac fever, a self-limited flu-like illness. Approximately 90% of LD cases are associated with infections by *L. pneumophila*. The most effective bacterial dissemination mechanism is through the spread of contaminated aerosols occurring primarily in condensers, cooling towers, showers, faucets, and hot tubs (Steinert et al., 2002; Wagner et al., 2007). Despite stringent water quality examinations, the formation of contaminated aerosols remains a crucial problem for disease spread (Fields et al., 2002).

*Legionella pneumophila* exhibit several modes of persistence in different environmental settings and in humans. Upon invasion of amoeba or human macrophages, *L. penumophila* form the *Legionella*-containing vacuole (LCV), a unique compartment with acquired components from early and late endosomes, mitochondria and the endoplasmic reticulum (ER), thus evading the bactericidal endocytic pathway and establishing a replicative niche (de Felipe et al., 2005; Isberg et al., 2009). The phagosome becomes a secure niche that supports the replicative phase of the bacteria. Importantly, hundreds of effector proteins synthesized by the Dot/Icm type IV secretion system of *L. pneumophila*. (Losick and Isberg, 2006; Abu-Zant et al., 2007; Price et al., 2011; Khweek et al., 2013; Abu Khweek et al., 2016). Like other intracellular bacteria such as *Coxiella* and *Chlamydia, L. pneumophila* alternate between a transmissive (virulent) and replicative (non-virulent) biphasic cycles to ensure bacterial survival in nutrient deprived or rich environments and transfer between different niches (Newton et al., 2010). In nutrient-rich environment, *L. pneumophila* enter the replicative phase and express few virulence factors. However, the switch to the transmissive phase is initiated in nutrient-limiting conditions, or when the phagosome is no longer supporting the replication phase of the bacteria. Increased motility, resistance to stressors, egress from the infected host and expression of several virulence factors are the hallmark characteristics of the transmissive phase (Newton et al., 2010). *Legionella pneumophila* is able to remain in the environment as free living planktonic bacteria or form bacterial biofilms that adhere to surfaces (Atlas, 1999; O'Toole et al., 2000; Mampel et al., 2006; Hindré et al., 2008; Stewart et al., 2012; Andreozzi et al., 2014). Moreover, *L. pneumophila* is able to differentiate into a mature intracellular form (MIF). Even though the MIF of *L. pneumophila* is inert and resembles cysts, it is extremely infectious (Faulkner and Garduño, 2002; Berk et al., 2008). Extracellularly, *L. pneumophila* enter into the viable non-culturable (VBNC) state which contributes to the resilience of this bacteria under different harsh environmental settings (Steinert et al., 1997; García et al., 2007) and hinder the detection of many *Legionella* species. Colonization and persistence in natural environment is mediated by biofilm formation (Valster et al., 2010), and survival within freshwater amoeba and *Caenorhabditis elegans* (Horwitz, 1983; Isberg et al., 2009).

Herein, we review several biological factors that contribute to biofilm persistence, the advantages that bacteria gain by being a member of the biofilm consortium and strategies to eradicate *L. pneumophila* biofilm.

## Multispecies and monospecies *L. pneumophila* biofilm

In freshwater environments, *L. pneumophila* is found as sessile cells associated with biofilms (Declerck et al., 2009; Declerck, 2010; Stewart et al., 2012). Biofilms allow the bacteria to attach to surfaces, or to be part of other bacterial communities. This can be attained by forming an extracellular matrix (ECM) that is composed largely of water, exopolysaccharides, proteins, lipids, DNA and RNA, and inorganic compounds (Costerton et al., 1987; Costerton, 1995; Sutherland, 2001; Shirtliff et al., 2002). Bacteria that are forming biofilm cycle between three developmental different phases. Stages of biofilm formation are initiated by attachments to a substratum, followed by maturation of the biofilm and formation of the extracellular matrix, then detachments and dispersion of the bacteria. During these phases, bacterial biofilms form three-dimensional structures that are separated by water channels, which allow entry of nutrients, oxygen, and discharge of waste products. Due to the complexity of biofilms that develop in natural environments, the behavior of *L. pneumophila* has mainly been tested in mono- or mixed species biofilms (Mampel et al., 2006; Piao et al., 2006; Hindré et al., 2008; Pécastaings et al., 2010; Stewart et al., 2012). Interestingly, *L. pneumophila* represent a minor species in freshwater and environmental biofilms, (Declerck et al., 2009; Declerck, 2010), and the occurrence of *L. penumophila* may be affected by other microorganisms in complex biofilms (Taylor et al., 2009). Some bacterial species positively promote the persistence of *L. penumophila* biofilm while others exhibit inhibitory effects (Stewart et al., 2012). Intriguingly, *Klebsiella pneumoniae (K. pneumoniae), Flavobacterium* sp., *Empedobacter breve, Pseudomonas putida*, and *Pseudomonas fluorescens* are among the bacterial species that positively contribute to the long-term persistence and presence of *L. pneumophila* in biofilms (Mampel et al., 2006; Vervaeren et al., 2006; Stewart et al., 2012). The authors reasoned that these species synthesize capsular and extracellular matrix materials which support the adherence (Kives et al., 2006; Basson et al., 2008; Wu et al., 2011), or provide the growth factors that stimulate growth of *L. penumophila* (Stewart et al., 2012). Other species antagonize the persistence of *L. pneumophila* within the biofilm such *Pseudomonas aeruginosa* (*P. aeruginosa*) (Stewart et al., 2012), *Aeromonas hydrophila, Burkholderia cepacia, Acidovorax* sp., and *Sphingomonas* sp. (Guerrieri et al., 2008). The inhibition could be due to the effect of *P. aeruginosa* homoserine lactone quorums sensing (QS) molecule on *L. pneumophila* biofilm (Mallegol et al., 2012), or production of bacteriocin (Guerrieri et al., 2008). Interestingly, *L. pneumophila* is able to persist in biofilm formed by *P. aeruginosa* and *K. pneumoniae* suggesting that the inhibitory effect of *P. aeruginosa* can be relieved by the permissive *K. pneumoniae* (Stewart et al., 2012). It is possible that *K. pneumoniae* provides the growth factors for *L. pneumophila* and at the same time dampens the inhibitory effect by *P. aeruginosa* (Stewart et al., 2012). Therefore, growth of *L. pneumophila* within biofilms is not only affected by the number and species of microorganisms present in the biofilm but also by the nature of interactions (commensalism or interference) between these organisms.

**Figure 1:**
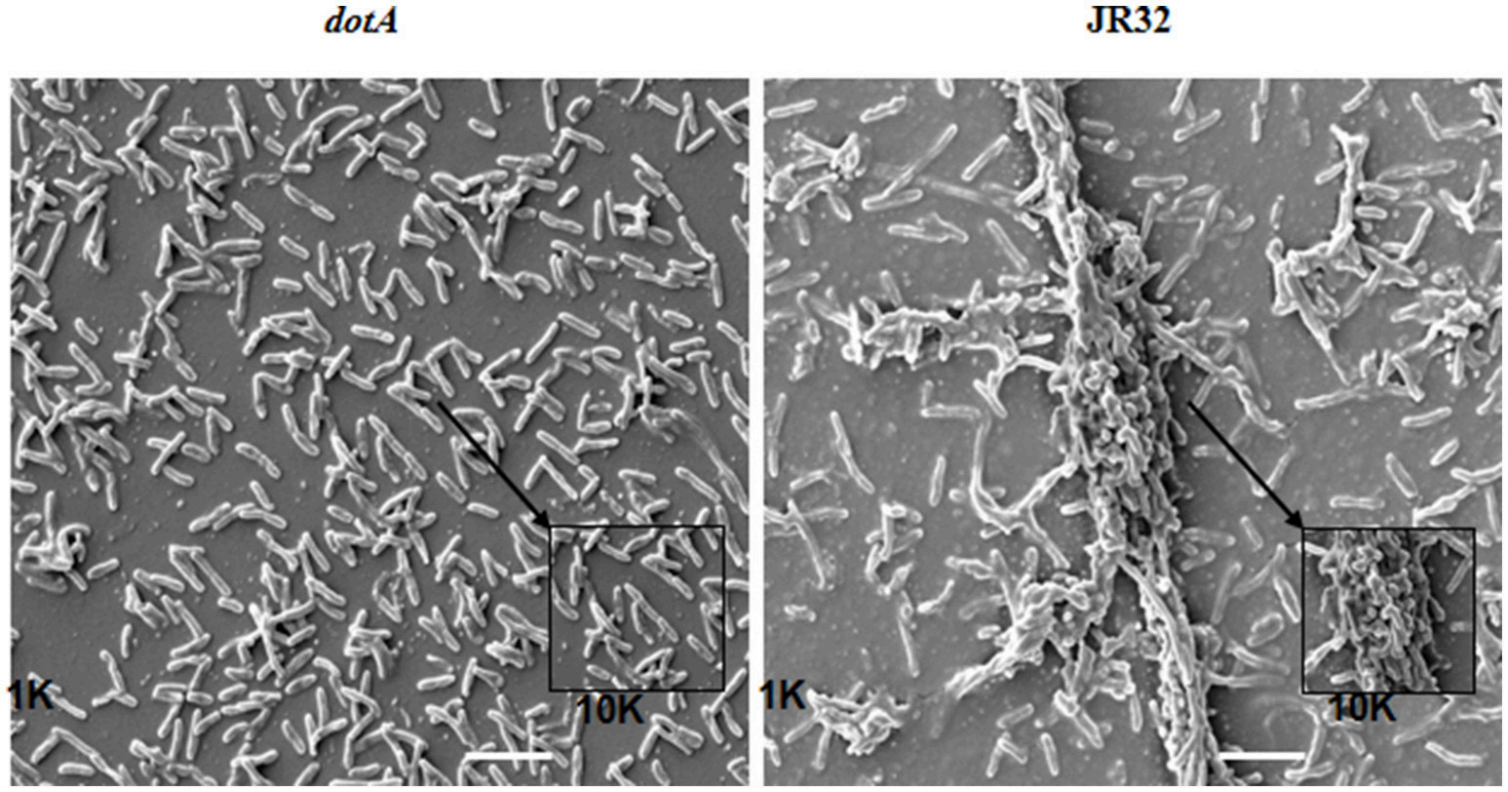
Scanning electron microscopy (SEM) of JR32 and *dotA* mutant. Larger images were captured with the 1000× objective lens while smaller images were magnified 10,000×, scale = 10 μm. The figure is adapted from Abu Khweek et al. (2013).

In the laboratory, *L. pneumophila* can form biofilm under stringent conditions in nutrient-rich Buffered Yeast Extract medium (BYE) under different temperatures (Mampel et al., 2006; Piao et al., 2006). The quantity, degree of adherence and rate of biofilm formation are correlated with different temperatures. At 25°C, *L. pneumophila* form mushroom-like biofilm that contain water channels. In contrast, *L. pneumophila* form thicker biofilm that lack the water channels at 37°C. However, the biofilm morphology of *L. pneumophila* grown at 42°C exhibits filamentous appearance with mat-like morphology. Furthermore, in the laboratory, we showed the ability of WT *L. pneumophila* to form biofilm when grown statically at 37°C for seven days as opposed to the *dotA* mutant that lacks the type IV secretion system (Figure 1).

Even though able to persist in multispecies biofilm, little is known about the factors encoded by *L. pneumophila* that mediates the attachment and persistence within biofilms created by other bacteria.

## Biofilms: a survival niche in oligotrophic environment

Biofilm is a rich environmental niche that harbors living and dead organisms as well as protozoa and other microflora. However, in a multispecies biofilm, the bacteria have to compete for the required nutrients to become an integrated member of the microbial community. Therefore biofilm-associated bacteria have to seek for the bacterial neighbors and the environment that best suits their growth and survival (Watnick and Kolter, 2000). *Legionella pneumophila* is exceptionally fastidious and require the mandatory supplementation of the laboratory media with amino acids and iron to grow (George et al., 1980; Edelstein, 1982). Therefore, survival and growth of *L. pneumophila* in oligotrophic environments is puzzling and indicates that the bacteria are able to utilize the essential nutrient from the bacterial community located in biofilms. Indeed, establishment of two-species and multispecies biofilms is one strategy by which *L. penumophila* overcome nutrients limitation in the environment. Therefore, adhering to a pre-established biofilm by other bacteria instead of attaching directly to the surface as a primary colonizer aids in *L. pneumophila* survival and incorporation in the biofilm community (Watnick and Kolter, 2000; Stewart et al., 2012).

Even though restricted to certain microbial species, necrotrophic feeding on the products of dead bacteria and tissues within the biofilm is likely the primary mode for deriving the required carbon, nitrogen, and amino acid for multiplication by *L. pneumophila* (Vervaeren et al., 2006; Taylor et al., 2009). Moreover, heterotrophic bacteria support growth of *L. pneumophila* on media that does not usually support growth because it is deficient in L-cysteine and ferric pyrophosphate (Wadowsky and Yee, 1983). Consistent with this, *L. pneumophila* show satellite colonies around some aquatic bacteria including *Flavobacterium breve, Pseudomonas* spp., *Alcaligenes* spp., and *Acinetobacter* spp. Further, *L. pneumophila* are able to obtain nutrients directly from algae and to grow on the extracellular products produced by cyanobacteria under laboratory conditions (Tison et al., 1980). Further, several algae such as *Scenedesmus* spp., *Chlorella* spp., and *Gleocystis* spp., support the growth of *L. pneumophila* in basal salt media (Declerck, 2010).

The second mechanism by which *L. pneumophila* obtain nutrient in biofilms is through amoeba. Protozoa serve as habitats that provide the environmental host for survival and replication of *Legionella* species in different environmental settings (Rowbotham, 1980; Newsome et al., 1998). Various amoeba such as *Acanthamoeba castellanii* can use *L. pneumophila* as a sole food source (Tyndall and Domingue, 1982), but also amoeba contribute to spread of *L. pneumophila* and protect the bacteria from various adverse effects such as antibacterial agents (Loret and Greub, 2010). Notably, persistence and adaptation of *L. pneumophila* in various amoebal hosts has been thought to contribute to pathogenesis of the bacteria. Intriguingly, biofilm colonization with *L. pneumophila* can be influenced by several species of protozoa (Rowbotham, 1981; Murga et al., 2001). Indeed, *L. pneumophila* can parasitize more than 20 species of amoebae, three species of ciliated protozoa and one species of slime mold (Kikuhara et al., 1994; Hägele et al., 2000). Further, it has been shown that multiplication inside the amoeba increased the capacity of *L. pneumophila* to produce polysaccharides and therefore enhanced its capacity to establish biofilm (Bigot et al., 2013). Further, *L. pneumophila* is able to grow off the debris from dead amoeba (Temmerman et al., 2006), and outbreaks of *L. pneumophila* are directly correlated with the biomass of protozoa. Moreover, in the absence of amoeba, biofilm-associated *L. pneumophila* numbers did not increase. Instead, bacteria were only able to persist in the biofilm community and in some cases entered the VBNC state in order to promote their survival (Declerck, 2010). Recently, increasing evidence suggests that metazoan such as the *C. elegans* could represent a natural host for *L. pneumophila* (Brassinga et al., 2010; Hellinga et al., 2015). It has been shown that *L. pneumophila* survive within biofilm containing protozoan and *C. elegans* (Rasch et al., 2016). Together, the ability to obtain nutrient in mixed species biofilms as well as to parasitize amoeba and *C. elegans* enhances the survival and persistence of *L. pneumophila*. The diversity of organisms in the biofilm consortium provide a diverse pool of nutrients for this fastidious organism.

## Factors that modulate *L. pneumophila* biofilm formation

### Role of Cyclic-di-GMP

Cyclic-dimeric diguanylate (c-di-GMP) is a bacterial second messenger that regulates several processes including bacterial pathogenesis and biofilm formation (Tamayo et al., 2007; Abu Khweek et al., 2010; Römling et al., 2013; Martinez-Gil and Ramos, 2017). Regulation of biofilm formation by c-di-GMP has been shown for several bacteria (Bobrov et al., 2011; Valentini and Filloux, 2016; Conner et al., 2017). Synthesis of the c-di-GMP is mediated by a GGDEF domain-containing diguanylate cyclases (DGCs) from two GTPs molecules (Simm et al., 2004) and degraded by an EAL-containing phosphodiesterases (PDEs) proteins (Simm et al., 2004).

The c-di-GMP signaling play important roles in the *L. pneumophila* life style (Levi et al., 2011; Allombert et al., 2014; Pécastaings et al., 2016). Interestingly, the *L. pneumophila* genome encodes for 22–24 GGDEF/EAL proteins, which vary between strains. Furthermore, overproduction of GGDEF/EAL proteins affect the ability of *L. pneumophila* to replicate within amoeba and macrophages and contribute to virulence of *L. pneumophila* (Levi et al., 2011; Allombert et al., 2014). In *L. pneumophila* Lens, three GGDEF/EAL-containing proteins have been shown to positively regulate biofilm formation (Pécastaings et al., 2016). Deletion of these proteins decreased biofilm formation without significant changes in the c-di-GMP level when compared to the wild type (WT) bacteria (Pécastaings et al., 2016). However, two GGDEF/EAL-containing proteins negatively regulate biofilm formation and deletion of these proteins resulted in overproduction of biofilm but surprisingly a decrease in the level of the c-di-GMP (Pécastaings et al., 2016). Therefore, GGDEF/EAL-containing proteins regulate biofilm formation by *L. pneumophila* in different mechanisms when compared to other bacteria.

Regulation of biofilm formation and the c-di-GMP activity in *L. pneumophila* has been attributed to the Haem Nitric oxide/Oxygen (H-NOX) binding domains family of haemoprotein sensors (Carlson et al., 2010). The H-NOX proteins are widespread in bacterial genomes and *L. pneumophila* is the only prokaryote found to encode two H-NOX proteins. Deletion of *hnox1* resulted in a hyper-biofilm formation phenotype without affecting growth in *pneumophila* in rich media (BYE), mouse macrophages or *Acanthamoeba castellanii*. Importantly, a GGDEF-containing protein is adjacent to *hnox1* and has been shown to exhibit diguanylate cyclase activity *in vitro* and when overexpressed, *L. pneumophila* results in a hyper-biofilm phenotype. The diguanylate cyclase activity is inhibited by the presence of the H-NOX in the NO-bound state; suggesting the regulation of the diguanylate cyclase activity by NO (Carlson et al., 2010). Exposure to NO resulted in increase in the biofilm intensity instead of dispersing the adherent bacteria. The excessive biofilm formation seems to be associated with a decrease in the level of c-di-GMP and the c-di-GMP degrading ability could enhance biofilm formation (Pécastaings et al., 2016). In the aquatic environment, *L. pneumophila* can be exposed to NO when it is in close contact of denitrifying bacteria. Further, *L. pneumophila* is exposed to NO produced by macrophages or protozoa. Therefore, NO sensing could regulate *L. pneumophila* biofilm formation.

### Role of iron

Iron is an essential nutrient and required for growth and replication of *L. pneumophila* (Reeves et al., 1981; Schaible and Kaufmann, 2004; Radtke and O'Riordan, 2006). The concentration of iron must be tightly controlled, as the excess of this metal can be toxic due to production of reactive oxygen species (ROS) (Andrews et al., 2003; Lemire et al., 2013). Importantly, high iron concentrations (a fivefold increase in iron pyrophosphate concentration) results in a strong inhibition of biofilm formation (Hindré et al., 2008). Furthermore, it has been shown that iron salts disturb biofilm formation of *P. aeruginosa* (Musk et al., 2005). Recently, the effect of iron pyrophosphate and several iron chelators on the persistence of *L. pneumophila* in mixed biofilm were tested (Portier et al., 2016). Addition of the iron chelator for ferrous iron, dipyridyl, DIP increased the quantity of bacteria regardless of the strain (WT or mutant in iron uptake). These data suggest a positive role for DIP in contributing to the persistence of *L. pneumophila* (Portier et al., 2016). Interestingly, DIP does not affect the bacterial population in biofilm or persistence of free-living amoeba in the biofilm and seems to be independent of iron acquisition systems as mutants in iron uptake were not affected by DIP. The authors hypothesized that DIP contributes to the persistence of *L. pneumophila* in biofilm by protecting the bacteria from the adverse effects of iron due to a decrease in ROS production (Portier et al., 2016).

### Genes involved in biofilm formation by *L. pneumophila*

Biofilm formation plays a role in the colonization, survival, dissemination and likely the pathogenesis of *L. pneumophila* (Lau and Ashbolt, 2009). However, little is known about the genetic factors and the molecular events involved in this process. Among the genes that have been shown to be required for biofilm formation is the putative twin-arginine translocation pathway which is required for transport of folded proteins across the cytoplasmic membrane. Insertional inactivation of the *tatB* and *tatC* genes inhibited biofilm formation by *L. pneumophila* (De Buck et al., 2005). Further, a strain lacking the flagellar sigma factor FliA (σ^28^) was found to be impaired for biofilm accumulation in static microtiter plates (Mampel et al., 2006). FliA is required for expression of genes associated with the transmissive phase of *L. pneumophila*, including flagella, macrophage infection, and lysosome evasion, as well as intracellular replication within *Dictyostelium discoideum* (Heuner et al., 2002; Molofsky et al., 2005). In mouse macrophages infection, biofilm-derived *L. pneumophila* down-regulate FliA expression compared to planktonic bacteria (Abu Khweek et al., 2013). Production of flagella is controlled by stationary phase regulatory network, sensing nutrient availability as well as the *L. pneumophila* quorum sensing (Lqs) signaling compound LAI-1(3-hydroxypentadecane-4-one) (Schell et al., 2016). Even though flagella has been implicated in biofilm formation by other bacteria, it has been shown that the flagella is not required for attachment and persistence of *L. penumophila* biofilm formed by *K. pneumonia* (Stewart et al., 2012). This is consistent with our observation showing the down-regulation of the flagella during biofilm formation in mouse macrophage (Abu Khweek et al., 2013).

The *Legionella* collagen-like (LcI) is an adhesin that binds to sulfated glucosamioglycans (CAGs) of the host extracellular matrix. This gene is widely distributed among different *L. pneumophila* environmental and clinical isolates but absent from other *Legionella* species that are rarely reported in patients and poor biofilm producers; suggesting that it was acquired by *L. pneumophila* through horizontal gene transfer (Duncan et al., 2011). Consistent with that, the GC content of *lpg2644* is different from the rest of *L. pneumophila* genome (Duncan et al., 2011). Further, mutation in this gene reduced biofilm formation, cell-cell adhesion and cell-matrix interactions (Duncan et al., 2011). This gene is differentially regulated during growth phases and biofilm formation (Mallegol et al., 2012). The regulation is mediated by *P. aeruginosa* quorum sensing (3OC12-HSL) during late stages of biofilm formation suggesting that the regulation may help in the dispersion of bacteria to reinitiate biofilm formation on another surface (Mallegol et al., 2012) which could be critical for the proliferation and dissemination of such waterborne pathogen (Lau and Ashbolt, 2009).

### Quorum sensing

In Gram-negative bacteria, quorum sensing (QS) regulates gene expression of various complex bacterial processes, including virulence, sporulation, bioluminescence, competence and biofilm formation (Zhu et al., 2002; Ng and Bassler, 2009). Importantly, the bacteria that exhibit QS signaling are usually identified in man-made water systems and it is now recognized that QS systems may play a role in the regulation of environmental biofilm production (Shrout and Nerenberg, 2012). *Legionella pneumophila* employ LAI-1 (3-hydroxypentadecane-4-one) QS autoinducer. This is the only (*Legionella* quorum sensing) Lqs system that has been described to date for *L. pneumophila* (Tiaden et al., 2007, 2008, 2010; Spirig et al., 2008). LAI-1 is produced and detected by the Lqs system and comprises the autoinducer synthase LqsA, the homologous sensor kinases LqsS and the response regulator LqsR (Tiaden et al., 2007, 2008; Spirig et al., 2008). It is not known if the Lqs system of *L. pneumophila* regulates biofilm formation. However, this system is homologous to the *cqsAS* QS of *Vibrio cholera*, which is involved in cell-density dependent regulation of virulence and biofilm formation (Miller et al., 2002; Zhu et al., 2002). The *P. aeruginosa* quorum sensing autoinducer (3-oxo-C12-HSL) inhibits biofilm formation of *L. pneumophila* (Mallegol et al., 2012). This effect is associated with down-regulation of the *lqsR* (Kimura et al., 2009). This suggests that QS could play a role in the dispersion of *L. penumophila* during later stages of biofilm development.

### *Legionella pneumophila* gene expression in biofilms

The first transcriptome analysis of *L. pneumophila* biofilm showed a substantial proportion of genes with differential gene expression compared to planktonic bacteria (Hindré et al., 2008). The gene expression pattern was compared with the replicative and transmissive phases during growth of *L. pneumophila* in *A. castellanii* (Brüggemann et al., 2006). Importantly, gene expression profile of sessile bacteria seems to resemble the replicative rather than the transmissive phase of *L. pneumophila*. This is supported by the well expression of genes involved in repression of the transmissive phase in sessile cells (Hindré et al., 2008), and suggests that biofilm is a suitable niche for *L. pneumophila* (Hindré et al., 2008). Among the genes that their expressions were highly induced in the sessile form are the *pvcAB* gene cluster which their expression is regulated by iron (Hindré et al., 2008). The *pvcA* and *pvcB* genes are homologous to the PvcA and PvcB proteins in *P. aeruginosa* and are required for the production of siderophore. In *L. pneumophila*, the *pvcA* and *pvcB* encode for a siderophore-like molecule, and might contribute to uptake and sequestration of iron below the toxic level. The second gene cluster, including *ahpC2* and *ahpD*, encodes for alkyl hydroperoxide reductases, displayed the highest induction in biofilm cells (Rocha and Smith, 1999). These proteins play a role in protection against oxidative stress (Rocha and Smith, 1999; LeBlanc et al., 2006). It is known that iron participates in the production of reactive oxygen intermediates and that the metabolism of iron and oxidative stress is related. Induction of both *pvcAB* and *ahpC2D* genes in sessile cells could thus be related and reflect the need for protection against oxidative stress resulting from high iron concentrations.

Further, the virulence of biofilm-associated *L. pneumophila* was assessed by examining the expression of the macrophage infectivity potentiator (*mip*) to transcriptionally active *L. pneumophila* infected in cell culture (Andreozzi et al., 2014). Expression of *mip* is important for intracellular replication in protozoa and human macrophages (Cianciotto and Fields, 1992). *mip* expression is down-regulated during early stages of infection but up-regulated in the last stages during escape from the host cell. Therefore, *mip* expression is up-regulated during the transmissive stages of *L. pneumophila* life cycle (Wieland et al., 2005). Expression of *mip* was constant at early stages of biofilm formation, when the bacteria did not require a new host for growth, which is similar to the replicative phase. In contrast, *mip* expression was predominately up-regulated at the end of biofilm formation which is similar to the transmissive phase *in vivo* (Andreozzi et al., 2014). The switch to the transmissive phase observed in planktonic form could be associated with *mip* up-regulation. This suggests that biofilm could protect the replicative form of *L. pneumophila*.

### *Legionella pneumophila* biofilm resistance, a challenge for biocides treatments

*Legionella pneumophila* is detected in environmental and artificial water systems as biofilms covering several environmental systems such as ventilation and conditioning systems (Lau and Ashbolt, 2009). In addition, biofilm-containing *L. penumophila* can become a transient or permanent habitat for other relevant microorganisms. Therefore, biofilm-associated organisms can survive for days, weeks or even months depending on the substratum and the environmental factors that stimulate biofilm formation (Blasco et al., 2008; Buse et al., 2014). To restrict *L. pneumophila* growth, numerous chemical, physical and thermal disinfection methods have been used against *L. pneumophila* (Kim et al., 2002). However, these treatments generally do not result in total elimination of the bacterium, and after a lag period, recolonization occur as quickly as the treatments are discontinued (Taylor et al., 2009). Biofilm-associated *L. pneumophila* is extremely resistant to disinfectants and biocides (Kim et al., 2002; Borella et al., 2005). Exposure of biofilm-encased bacteria to biocides could lead to entry into a VBNC status (Giao et al., 2009). Chlorine and its derivatives are the most common biocides used in disinfection protocols and have been shown to be appropriate in eliminating planktonic *L. pneumophila* but not biofilms (Cooper and Hanlon, 2010). Resistance of *L. pneumophila* to disinfection is due not only to its capacity to survive within biofilm, but also the bacteria exhibit the intra-amoebal life-style (Steinert et al., 1998; Hilbi et al., 2011). Therefore, amoeba- associated *L. pneumophila* are more resistant to disinfection possibly due to differences in membrane chemistry or life cycle stages of this primitive organism (Taylor et al., 2009; Dupuy et al., 2011). Vesicles containing intracellular *L. pneumophila* released by amoeba are resistant to biocide treatments (Berk et al., 1998). Notably, these vesicles remained viable for few months (Bouyer et al., 2007). Understanding the molecular mechanisms that governs the intra-amoeba related resistance should pave the way for development of new strategies to eradicate *L. pneumophila*.

Other methods have been used to limit *L. pneumophila* such as applying heat which has been shown to be effective in reducing the number of bacteria and protozoan trophozoites, but infective against killing cysts (Storey et al., 2004; Farhat et al., 2012). UV radiation is also effective when the bacteria are in direct contact with the radiation (Schwartz et al., 2003). However, higher UV intensities are required to inactivate the protozoa (Hijnen et al., 2006). Other methods have been proposed to control *L. pneumophila* growth such as controlling the carbon source within anthropogenic water system (Pang and Liu, 2006), or addition of phages to control bacterial or specifically *L. pneumophila* growth. The phage is capable of degrading polysaccharides and therefore destabilizing the biofilm (Hughes et al., 1998; Lammertyn et al., 2008). In addition, nanoparticles have been shown to be effective in reduction of *L. pneumophila* biofilm volume and showed some efficacy against *Staphylococcus aureus* and *Staphylococcus epidermidis* biofilms (Subbiahdoss et al., 2012; Taylor et al., 2012; Raftery et al., 2014). Several natural compounds (biosurfactants, antimicrobial peptides, protein and essential oil) have been shown to exhibit anti-*Legionella* properties (Berjeaud et al., 2016). Collectively, it is necessary to control *L. pneumophila* growth and their natural hosts to optimize eradication of the bacteria.

## Conclusion

**Figure 2:**
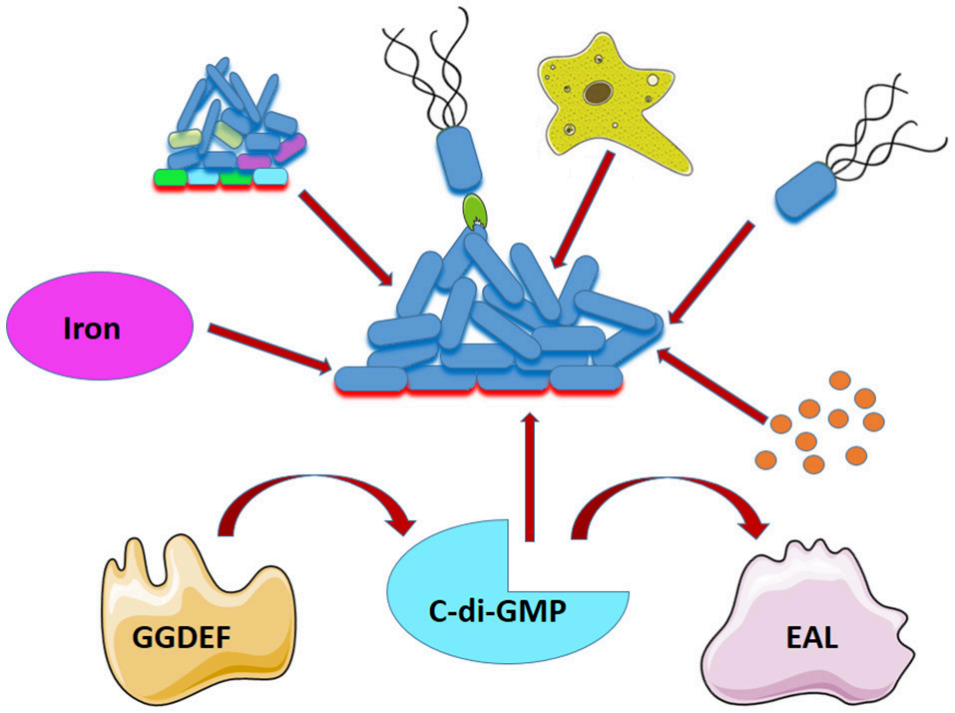
Factors that affect *L. pneumophila* biofilm formation. Association of *L. pneumophila* in biofilm is affected by presence of other bacteria (mixed colors), adhesin (green) presence of amoeba (Yellow), flagella (black), iron (magenta), quorums sensing (orange), and the level of C-di-GMP which is affected by a GGDEF and EAL-containing proteins.

Several chemical and physical parameters can influence the behavior of *L. pneumophila* in biofilms, including the surface, the temperature, carbon and metal concentrations, and the presence of biocides (Wright et al., 1991; Bezanson et al., 1992; Rogers et al., 1994; Donlan et al., 2005; van der Kooij et al., 2005; Liu et al., 2006; Mampel et al., 2006; Pang and Liu, 2006; Piao et al., 2006; Lehtola et al., 2007; Türetgen and Cotuk, 2007; Hindré et al., 2008). Biological factors such as being a member of mixed species biofilm or parasitizing free-living amoeba or nematodes influence biofilm formation by *L. pneumophila* (Figure 2). Biofilm- associated *L. pneumophila* is resistant to biocides and Legionellosis outbreaks have been attributed to biofilms. Therefore, it is essential to design new remedies for eradication of *L. pneumophila* biofilm in different environmental settings. Treatment studies should be performed when the bacterium is in its natural host to determine how the bacteria are protected inside the amoeba and if the passage through the natural hosts modify the resistance. Thus, preventing biofilm formation appears as one strategy to reduce water system contamination.

## Author contributions

AAK wrote the review and AA edited the manuscript.

### Conflict of interest statement

The authors declare that the research was conducted in the absence of any commercial or financial relationships that could be construed as a potential conflict of interest. The reviewer EC and handling Editor declared their shared affiliation.
